# Smoke Alarms for Informal Settlements: Monitoring and Challenges from a Large-Scale Community Rollout in Cape Town, South Africa

**DOI:** 10.1007/s13753-022-00457-8

**Published:** 2022-11-29

**Authors:** Robyn Pharoah, Patricia Zweig, Richard Walls, Rodney Eksteen

**Affiliations:** 1 Research Alliance for Disaster and Risk Reduction (RADAR), Stellenbosch University, Stellenbosch, 7602 South Africa; 2 Fire Engineering Research Unit (FireSUN), Stellenbosch University, Stellenbosch, 7602 South Africa; 3 College of Engineering, Architecture and Technology, Oklahoma State University, Stillwater, OK 74078 USA

**Keywords:** Fire safety, Informal dwellings, Smoke alarms, South Africa

## Abstract

This article presents the findings of a pilot project to test the large-scale rollout of smoke alarms in an informal community in Cape Town, South Africa. The work provides novel insight into the effectiveness and challenges associated with using smoke detectors in low-income communities. Technical details and detector considerations are also provided that will assist in enhancing future interventions. The project installed 1200 smoke detection devices in TRA informal settlement in the suburb of Wallacedene, in the City of Cape Town, and monitored their effectiveness for a period of 12 months. The monitoring showed that there were 11 real activations, where the presence of the devices likely saved lives and homes. The project also identified a series of challenges, especially in relation to nuisance alarms, where everyday household emissions, dust, and insect ingress caused false alarms, leading some participants to uninstall devices. The findings of the pilot study suggest that although smoke detectors could provide a valuable tool for reducing the frequency and impact of informal settlement fires in South Africa and elsewhere, they need to be adapted to meet the specific needs and conditions encountered in informal dwellings. Modifications, such as adjusting device sensitivity, preventing dust and insect ingress and tailoring devices to everyday conditions, will be essential to make smoke alarms more suitable and effective in the future. Smoke alarms could become an important component of low-income community fire safety if such challenges can be addressed.

## Introduction

Uncontrolled informal dwelling fires are a significant threat to the lives and well-being of poor communities across the Global South (Rush et al. 2020), where over 1 billion people live in densely populated informal settlements (United Nations 2022, p. 18). Dwelling fires cause more than 150,000 deaths annually, with over 95% of deaths and burn injuries occurring in low and middle-income countries (WHO 2018). Fires are increasingly prevalent in densely populated urban settings where informality, high population densities, and poverty create a physical and social environment conducive to ignition and spread. The risk of fires is particularly high in informal settlements, but fires also pose a threat in other contexts. In September 2020, for example, a fire swept through one of Greece’s largest migrant camps on Lesbos, destroying nearly 13,000 people’s dwellings (BBC News 2020). In addition to avoidable deaths, injuries, and trauma, such fires further impoverish already socioeconomically vulnerable households (Pharoah 2009; Raphela 2011; Twigg et al. 2017; Walls, Cicione et al. 2019; Rush et al. 2020), signaling an urgent need for interventions to reduce the incidence of fires and protect lives and property.

In South Africa, firefighting, disaster management, infrastructure repair, burn victim hospitalization, and humanitarian relief cost municipalities millions each year (Maritz et al. 2012). In 2017, a large fire in an informal settlement called Imizamo Yethu in Cape Town, for instance, officially cost the city at least USD 7 million, although Kahanji et al. (2019) estimate that the true cost may have been as high as USD 14 million when all infrastructure-repair costs are included. A fire in another informal settlement, Masiphumelele, in 2020, was declared a disaster and cost the national government USD 4.7 million in relief costs (South African Government 2020). These figures exclude numerous indirect costs, and do not reflect the magnitude of material, physical, and mental health costs suffered by poor households affected by the fires. Reducing the incidence of fires, and their associated human, development, and financial implications, thus represents an important avenue of disaster risk reduction in the city. This approach is in keeping with the global shift in emphasis from disaster preparedness, response, and humanitarian assistance to proactive work to disrupt risk accumulation processes and prevent disasters (Lazarevski and Gjorgon 2017; Wisner 2019).

Global research suggests that early detection of home fires is critical in preventing deaths and injuries (Bukowski 2001). It is estimated that smoke inhalation injuries cause 50% to 80% of fire deaths (Hall 2011). Smoke also often incapacitates and disorients people so that they cannot escape (Murphy 2011), with sleep studies showing that people do not commonly wake up when they smell smoke (SFPE 2019). In addition to CO, toxic gases released by synthetic materials, such as upholstered furniture, are the most common cause of death during fires (Stec 2017). Poor ventilation in informal dwellings also increases the risk of asphyxiation associated with smoldering fires (Walls et al. 2020). Thus, if a potential fire can be detected during the smoldering stage, before flames appear, this reduces the risk of asphyxiation and provides time and an opportunity to escape or extinguish fires before they burn out of control, thus preventing deaths and injuries, and reducing the spread and impact of fires (Zweig et al. 2018).

Smoke alarms could potentially reduce the incidence of fires in informal dwellings and save lives and property, and represent a powerful tool to reduce the risk of large and small-scale fire disasters. Smoke alarms have been shown to provide one of the most cost effective ways of reducing the impact of fires internationally (Warmack et al. 2012). Alarms are used in formal buildings around the world, and are mandatory in many countries (Warmack et al. 2012; Ahrens 2021), but, until recently, they have not been used in informal housing.^1^

Work in South Africa is pioneering the application of alarms in informal environments. The prospect of preventing fires has seen several projects to install alarms in communities, particularly in the Western Cape Province, and especially Cape Town. The provincial Western Cape government has spent millions of South African Rand^2^ on installing close to 12,000 smoke alarms in informal settlements in the province (du Toit 2021). The City of Cape Town metropolitan municipality, sponsored by a large insurance company, has installed 1,200 devices in high-risk areas in the city (Knight and Chan 2019). Nongovernmental organizations, such as the American Red Cross, in collaboration with the Red Cross in South Africa, have also run pilot projects in Cape Town. Outside of South Africa, the American, South African, and Kenyan Red Cross organizations have conducted trials on the use of smoke alarms in Nairobi, Kenya (Eli 2015). In addition to smoke alarms, a South African company, Lumkani, has installed over 20,000 fire alarms in informal settlements in South Africa (GSMA 2021), and is widely recognized in South Africa and internationally for its package of alarms and low-cost insurance cover for people living in informal settlements.

The problem with these programs is that the unique challenges associated with using alarms in informal environments have not been adequately interrogated, and there is insufficient data on alarm performance over time. Few studies have documented the use and challenges associated with detector technologies in informal settlements, or other low-income communities such as refugee camps.

This article presents findings from a pilot project undertaken in the City of Cape Town (CoCT), in the Western Cape in 2017. The article first outlines fire risk challenges in Cape Town and the social context in which that risk is rooted. It then presents general fire safety aspects and considerations for designing fire-detection devices for informal dwellings. The project is then introduced, looking at how it was rolled out and feedback was received during the monitoring period. The work suggests a need for caution in rolling out installations without a sufficient understanding of their performance in the uniquely challenging environment found in informal dwellings.

## Fire Incidence in Cape Town

**Fig. 1:**
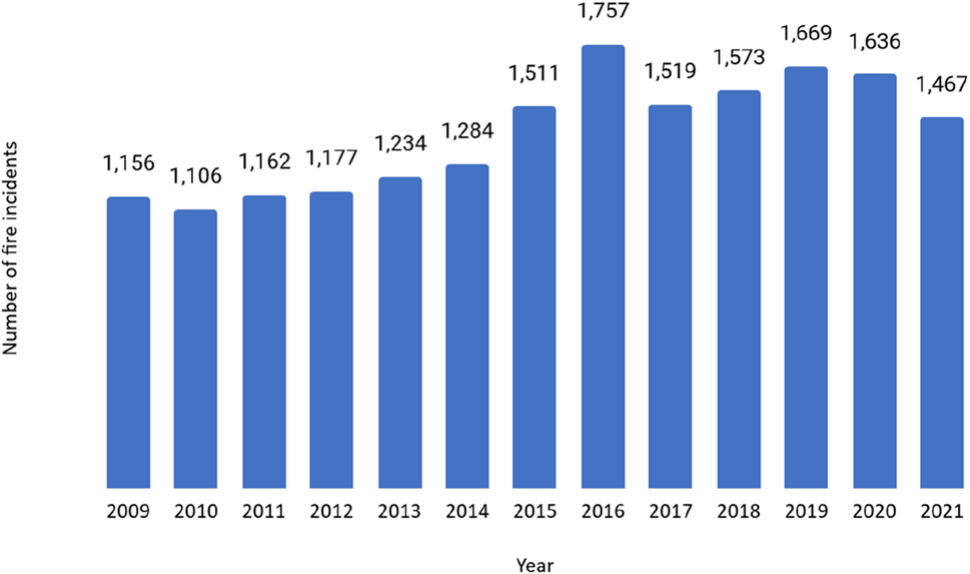
Fire incident logs for informal dwelling fires in the City of Cape Town, January 2009−October 2021. *Source* City of Cape Town (CoCT 2009–2021).

South Africa has some of the world’s largest informal settlements, with many in the CoCT (Kahanji et al. 2019). Statistics South Africa (2020) estimates that approximately 13% of households in South Africa, or around 2.2 million people, lived in informal dwellings in 2019, with numbers in the Western Cape and Gauteng Provinces significantly higher (both 19%).^3^ Another three million households live in “backyard shacks” in low-income formal areas. In Cape Town, fires occur almost every day (Walls and Zweig 2017), and the city has by the far the highest recorded number of informal settlement fires in South Africa (Zweig et al. 2018; Walls, Cicione et al. 2019). However, the national data may be incomplete for other large metropolitan areas (for example, Johannesburg, Ekurhuleni, and Tshwane), which may experience just as many, or possibly even more fires. As shown in Fig. 1, incident logs recorded by the CoCT’s Fire and Rescue Services show that they responded to 18,251 informal dwelling fires between January 2009 and October 2021 (CoCT 2009–2021). For the most part, these affect a handful of dwellings, but sometimes they sweep through entire settlements, and can spread with devastating speed (Mccullough 2017; Rush et al. 2020; Walls et al. 2020). In January 2013, for example, a fire in Khayalitsha (one of Cape Town’s largest low-income areas) left five dead and 4000 homeless (Sacks 2013). A fire in Masiphumele in December 2015 killed one person and left 1000 homeless (Walls and Zweig 2017), while the fire in November 2020 left two dead and displaced 4000 (Pitt and O’Regan 2020). The notorious fire in Imizamo Yethu in 2017 killed four people, destroyed 2194 dwellings, and left almost 10,000 people homeless (Kahanji et al. 2019).

## Fire Risk Context in Cape Town

The complex fire risk accumulation processes at play are embedded in a broader context of acute poverty in low-income areas and socioeconomic marginalization of the urban poor. Marginalization processes especially affect those living in the sprawling informal settlements that have become a ubiquitous feature of South Africa’s urban landscape (Pharoah 2009). As in South Africa’s other cities, this is rooted in both apartheid planning, which banished “Black” South Africans to the marginal urban edge, and the continued “peripheralization” of informal settlements and low-income housing in the post-apartheid era (Horn 2020; Huchzermeyer 2003). Despite the government’s commitment to spatially and socioeconomically integrating South Africa’s cities since 1994—primarily through the rollout of an extensive subsidized housing program for poor households—housing backlogs and the failure of housing policy to keep pace with population growth have led to growth in the number and density of informal settlements in the city (Pharoah 2009). This trend has been amplified exponentially by the economic hardships created by the COVID-19 epidemic. Local governments are notoriously under-resourced (Pieterse 2009) and struggle to provide services, but informal settlements also occupy an ambiguous legal and political space. While legislation grants de facto tenure rights and the right to basic services to people who are living on land not legally their own, the government is often prevented from providing services such as electricity, piped water, and fire hydrants without the consent of titular landowners. The South African government has also identified informal settlements as a key obstacle to national development (NPC 2012), and has prioritized their elimination, which also often discourages service provision.

**Fig. 2:**
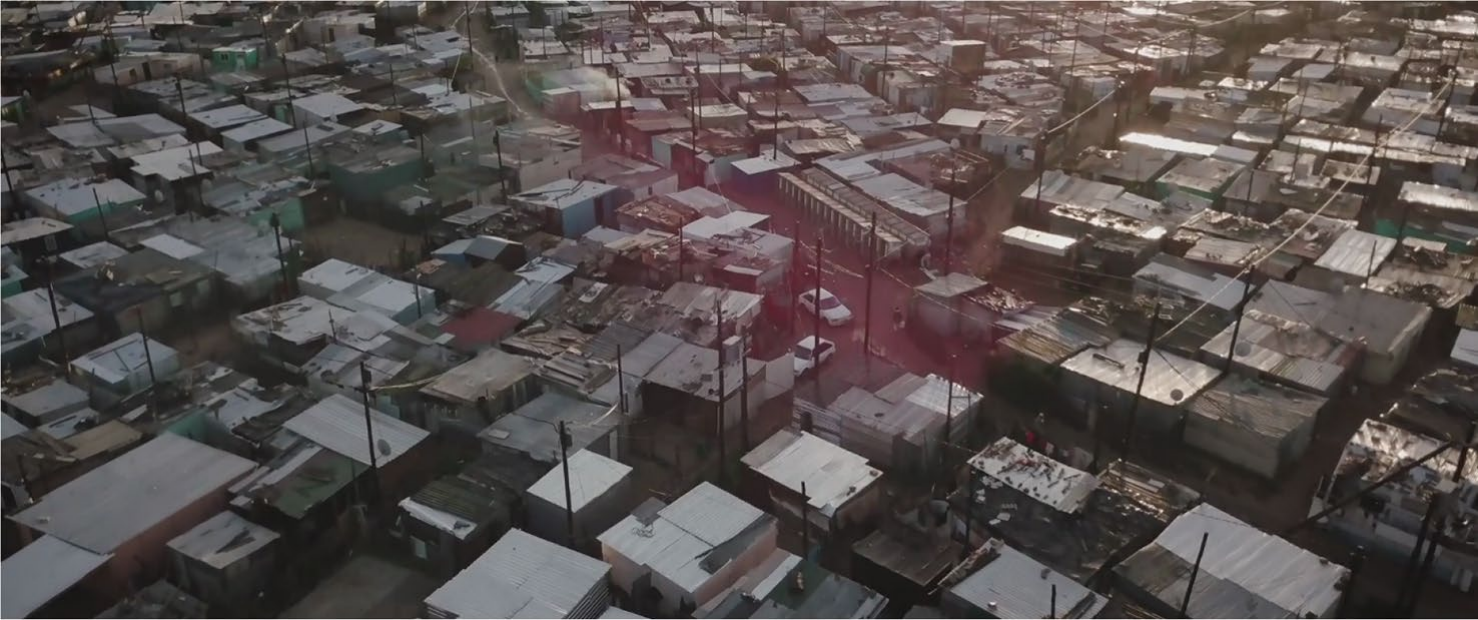
Aerial view showing a dense settlement in Cape Town. 2020. Image used with permission of Justin Sullivan.

Informality brings together fuel and flame. Dwellings in informal settlements and backyards are usually built and insulated using flammable materials like wood, plastic, and cardboard. At the same time, people rely on cheap but hazardous sources of energy and light, such as candles, paraffin stoves, and open fires (Pharoah 2009; Walls and Zweig 2017; Cicione et al. 2019). Fires are also frequently caused by electrical faults owing to overloaded plug points and illegal electricity connections (Morrissey and Taylor 2006; Zweig et al. 2018; Walls, Eksteen et al. 2019). High residential densities increase the risk of fires spreading and make them more difficult to reach and extinguish (MacGregor et al. 2005; Morrissey and Taylor 2006; Walls, Eksteen et al. 2019), while the storage of flammable materials between houses facilitates fire spread (Walls, Eksteen et al. 2019). This problem is compounded by inadequate basic services and infrastructure such as piped water and fire hydrants that could help in putting out fires (Pharoah 2009; Cicione et al. 2019; Kahanji et al. 2019). Figure 2 shows an aerial view of a typical, relatively dense, urban informal settlement in Cape Town, which illustrates a number of the issues discussed above.

Fires are also strongly linked to social conditions (Van Niekerk et al. 2009; Gevaart-Durkin et al. 2014). For example, research suggests that fires are often associated with heavy drinking, when people knock over candles and stoves or fall asleep with something burning (Morrissey and Taylor 2006; Van Niekerk et al. 2009; Twigg et al. 2017; Zweig et al. 2018) and stem from limited childcare options that result in children being left alone to cook and engage in other dangerous activities (DiMP 2002; MacGregor et al. 2005; Morrissey and Taylor 2006; Solomon 2006). Fires often occur late at night, when people are sleeping (Zweig et al. 2018). Once ignited, it can be difficult to escape from dwellings, particularly where shacks lack windows, or have security gates and window bars (Zweig et al. 2018).

There is very little data on the impact of fires, but experience suggests that events have a profound effect on households. In addition to deaths and injuries, already poor households often lose everything they own and struggle to recover. This includes loss of important documents such as identity books or cards and birth certificates, which are needed to access state-support grants and other safety nets (Pharoah 2009). A small number of studies also show that there are psychosocial costs. Households not only lose belongings, but also community, familial, and social ties, as well as connections and self-respect, especially where they are displaced for a long term or need to rely on help from the state or others (Stewart 2008; Stewart et al. 2012). Some studies also highlight trauma (Raphela 2011; Rush et al. 2020) and the psychological effect of personal and group stress and social fragmentation following fires (Stewart 2008; Stewart et al. 2012).

## Fire Safety

Given the profound impacts on poor households, reducing the risk of fires is essential. Owing to the challenging conditions in informal settlements, fire-safety interventions have been confined to formal environments historically, but ongoing research in the Western Cape is identifying measures to improve fire safety in informal environments. This section examines a framework for integrated fire safety in informal settlements, and the conditions in informal settlements and dwellings that must be considered in designing smoke or fire detection devices.

### Fire Safety for Informal Settlements

The provision of fire safe homes and infrastructure is described in national legislation globally (Republic of South Africa 1977) and in the constitution of many countries. Formal fire engineering codes of practice provide guidelines for safety distances, materials, building layouts, suppression systems, and many other aspects (SABS 2020). Informal settlements, in contrast, are defined by their lack of compliance with formal codes of practice, and municipal infrastructure requirements and guidelines for formal housing cannot be applied or enforced in these communities. However, a fire safety guideline for informal settlements has been developed recently to guide communities, fire departments, municipalities, nongovernmental organizations, and other entities seeking to address safety in South Africa’s informal settlements (Walls et al. 2020).

**Fig. 3:**
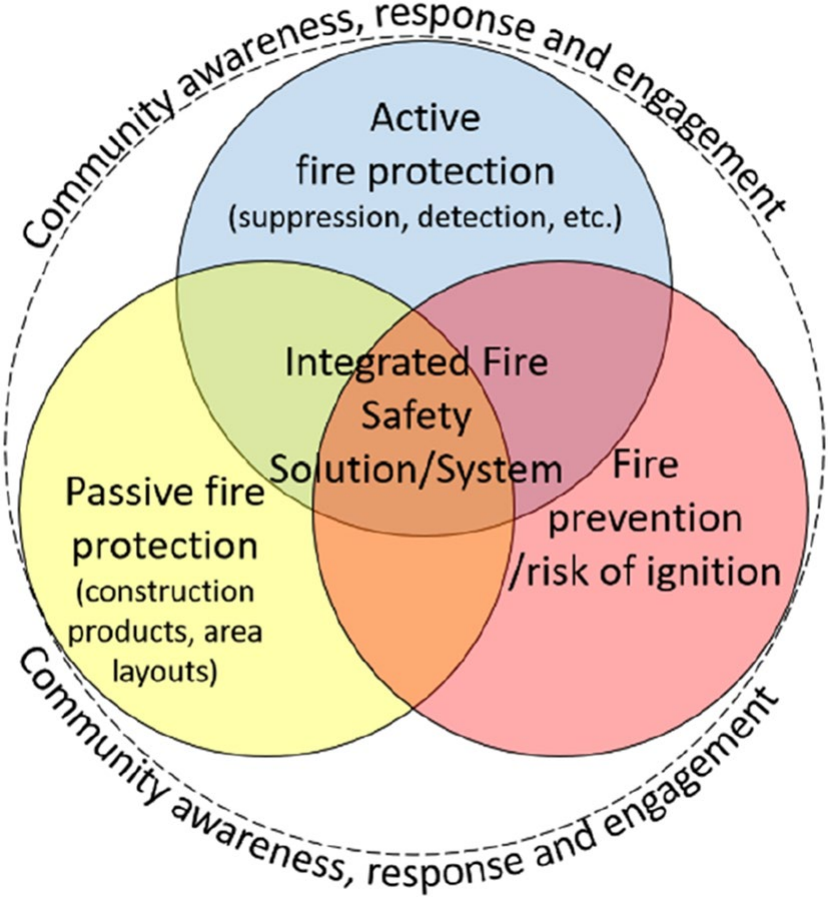
Framework for improving informal settlement fire safety. *Source* Walls et al. (2020).

As presented in Fig. 3, an integrated fire safety solution requires a basket of interventions. These include active protection systems (for example, fire suppression measures), passive protection methods (for example, noncombustible walls), and fire prevention (for example, safe electrification). Combining approaches can help to reduce the number of fires starting and spreading, and enable fires to be more readily extinguished. Detection systems, such as fire and smoke alarms, fall within the active protection category as they require a response by people when activated. Engineered measures alone, however, will not address fire risk. It is essential that interventions are paired with community-level work that engages at-risk populations. This includes, but extends beyond, awareness raising and equipping people with the skills and resources needed to prevent and respond to fires when they occur. In keeping with decades of experience in the development sector (Chambers 1994, 1997; Keare 2001; Cornwall 2006; Flint and Blyth 2021) and community-based disaster risk reduction (Maskrey 1989; Twigg 1999; Heijmans 2009; Maskrey 2011; Shaw 2012), it is critical to engage those affected in the design and implementation of fire safety measures.

### Detection Considerations

**Fig. 4:**
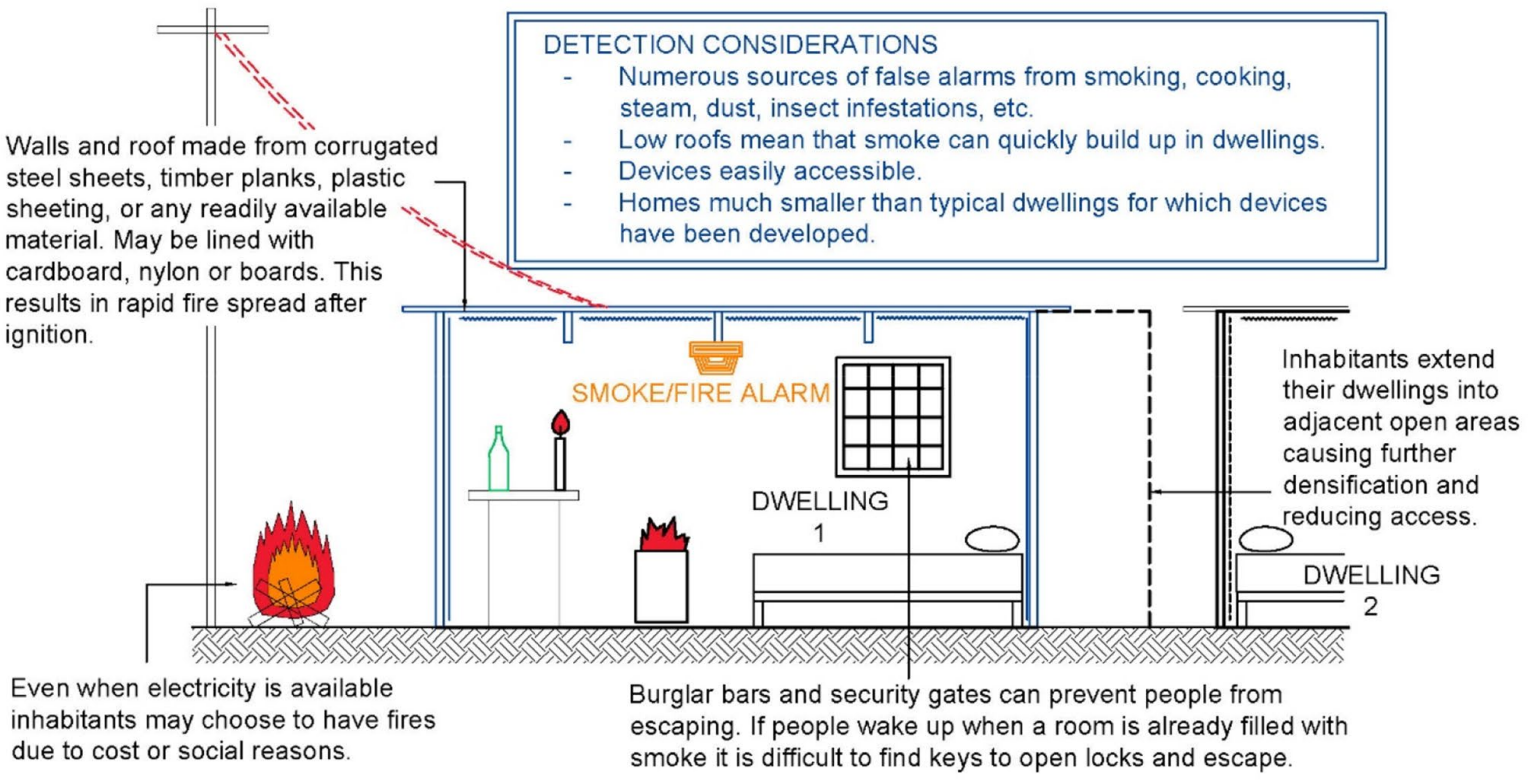
Conditions in informal settlements that can contribute to fires in South Africa

Figure 4 depicts several important considerations to be factored into the design of fire detection devices in informal settlements. Dwellings are generally small (2−8 m in either width or breadth) and have low roofs (1.8−2.5 m), making them smaller than the North American or European dwellings for which detectors have typically been developed. Furthermore, even within a small dwelling there may be several subdivisions to create rooms or compartments for different family members, or even different families. Room dividers and wall linings may be made from cardboard, nylon, curtains, timber, or other materials, most of which promote rapid fire spread after ignition. Hence, residents have very little time to respond between ignition and when conditions become fatal. Because electricity is expensive, even where electricity is available, households also often continue to use flaming sources of energy, including paraffin stoves, candles, and open fires, both inside and outside dwellings.

Smoke or fire detectors may be fixed to either the roof/ceiling or cross beams supporting the roof. These devices are typically exposed to emissions from daily household activities such as cooking, open fires (outside or inside), and bathing; dust from earthen floors and unpaved paths outside; smoking; and lighting, such as the use of paraffin lamps. Insects are relatively common in these settlements, influenced by factors such as lack of refuse removal or the presence of standing water. Insects may infiltrate devices, causing them to malfunction.

Devices are readily accessible, which is both a benefit and a challenge. If there is a false alarm, most devices have a “hush” or “silence” button to deactivate the device. Residents can quickly access and silence the device, and if devices become dirty or if there is a build-up of dust, residents will be more likely to notice it and can remove and clean them. Yet the same low ceilings that guarantee access also increase the buildup of particulates and mean that devices can be more easily bumped or damaged. Where alarms use typical 9V batteries, these may be removed for use in other devices such as television remotes.

There are two main lower-cost detector technologies considered suitable for informal settlements in South Africa: smoke alarms activated by smoke particles or fire alarms activated by rising temperatures (rate-of-rise alarms). The former activate during the smoldering phase of a fire, before flames appear, such as where a cigarette falls onto a couch or bedding. There is little heat released at this stage but there may be enough smoke to cause death by asphyxiation—particularly where people are asleep and unable to extinguish a fire. Fire alarms, on the other hand, are activated by the heat associated with flames (Zweig et al. 2018). Smoke alarms are the focus of this article. Fire alarms typically experience far less nuisance alarms as they only activate when either the rate of temperature change or a specific temperature threshold is reached.

## The Smoke Alarm Project

This section provides an overview of the smoke alarms project, the testing and selection of the devices for the pilot study, and the methodology used to evaluate their performance, as well as the challenges encountered.

### Overview and Background

With the discussions above as the context, the Western Cape’s Disaster Management Centre (WCDMC) Fire and Rescue Services Directorate has been working with various partners to develop strategies to reduce fire incidence and prevent fire-related injuries and deaths in the province. In 2016, the Directorate initiated a smoke alarm program to combat fires in informal settlements across the Western Cape. The first step was to test the use of smoke alarms in informal settings and the specific issues and constraints associated with their use in informal dwellings. The two-phase pilot project sought to determine the most suitable type of alarm for testing, installed high-performing alarms in a trial community, and monitored their use over a 12-month period (Zweig et al. 2018), with the second phase being the focus of this work.

The collaborative project involved four stakeholders. The core project team comprised the WCDMC’s Fire and Rescue Services Directorate, the Research Alliance for Disaster and Risk Reduction (RADAR), and the Fire Engineering Research Unit (FireSUN), the latter two both based at Stellenbosch University, as well as the community leadership of the trial community. These partners brought together technical knowledge, expertise on fire behavior and fire risk drivers in informal settlements, as well as social research, monitoring, and evaluation. A private insurance company, Santam, supported the project financially.

In this respect, the project demonstrated an interdisciplinary collaboration of researchers from very different research paradigms and community members, all working together effectively to solve a real-world problem. It represents what Okada et al (2018, p. 429) referred to as “implementation science”: the coproduction of knowledge and codesign of context-appropriate solutions that bridge gaps between disciplines, and recognizes the capabilities and resources available to those affected by, and responsible for, addressing complex problems in a risk governance context. It also reflects the increasing global shift away from traditional state-centric methodologies towards more inclusive community-led and collaborative approaches (Eversole 2012; Gibson and Wisner 2016; Duda et al. 2020), which is underscored throughout global agreements such as the Sendai Framework (Kelman 2017).

### First Phase: Engineering Testing

The first phase entailed fire-engineering testing in laboratory and field conditions (see Walls et al. 2017). The project conducted full-scale shack tests on a photoelectric smoke alarm, an ionization smoke alarm, and a rate-of-rise heat detector to determine activation times under both smoldering and flaming fire conditions.^4^ Nuisance alarm tests were also conducted to investigate how the alarms performed when exposed to smoke and steam from cooking, flaming energy sources like candles, and traditional home heating methods. Ionization-type detectors detect smaller particles and flaming fires more readily, whereas photoelectric devices are more suitable for smouldering fires, which are associated with large smoke particles. Hence, from a life-safety perspective photoelectric devices were considered more suitable for informal dwellings, where early detection is important.

Consistent with the literature, the testing showed that photoelectric smoke alarms performed best overall. These activated fastest in the smoldering fire tests, while the rate-of-rise detectors did not activate at all. In the flaming fire tests, the ionization alarms activated slightly faster than the photoelectric alarms, but they were less prone to nuisance activations. The rate-of-rise detectors only activated shortly before flashover (that is, when a room is engulfed in flame), which would provide insufficient time for occupants to escape unharmed (Walls et al. 2017). Given that flashover can occur in less than one minute in informal dwellings and the danger posed by smoke-inhalation, particularly when people are asleep, fires should be detected as early as possible, in the smoldering phase. The photoelectric alarm, with a 10-year tamperproof battery and silence function, was selected for the community-level trial.

### Second Phase: Community Testing

In the second phase, 1,200 alarms were installed in a trial community and their effectiveness was monitored over a 12-month period. A team of community members was trained to install the alarms. Each household was shown how to use the device, test it regularly, and activate the silence/hush feature in the event of false alarms. Given the objective to reduce inhalation deaths when people are sleeping, the devices were installed on the roof, in the primary sleeping area. The RADAR group also trained a team of young unemployed women to undertake the monitoring survey that followed. This survey recorded the frequency and causes of alarm activations, people’s views about the alarms, and their effectiveness in enabling people to escape or put out fires. The installation team initially installed alarms under the guidance of the WCDMC, but later took full ownership of the process.

TRA informal settlement was temporary relocation area for fire-displaced citizens that has since become permanent, and took on the name. It is located in Wallacedene on the eastern fringe of Cape Town, and was selected as a case study site because it had established a track record of working with external partners in carrying out collaborative risk reduction initiatives (RADAR 2015). At the time, the TRA was home to between 4000 and 5000 people, living in approximately 1400 informal dwellings, meaning that alarms were installed in approximately 85% of the homes in the settlement. As in many other informal settlements, the residents did not have access to formal electrical infrastructure historically, and use of paraffin stoves and open flames, as well as potentially dangerous illegal electricity connections, were associated with frequent fires (RADAR 2015)—although there is no reliable, settlement-specific fire incident data available to determine an exact number. The trial coincided with the city connecting the settlement to the formal electricity grid.

The initial intention was that the small monitoring team would conduct a monthly door-to-door survey to collect quantitative data on activations during the month. Although the surveys were scheduled to continue for a six-month period, they were discontinued after two months because many community members resented being interviewed repeatedly, and the survey team became concerned about their personal safety when visiting dwellings alone—a problem that could perhaps have been avoided if the monitoring component was better explained on installation. A new, less formalized qualitative monitoring program followed, undertaken by project staff. This involved visiting households reporting alarm activations and interviewing household members and their neighbors about the circumstances of these activations, as well as ad hoc interviews with other residents about their experiences with the alarms.

## Project Outcomes and Insights

This section presents the findings of the monitoring research, including the suggested impact of the alarms in reducing fire incidents in the settlement, the reception by the community, and the performance of the devices over the 12-month monitoring period.

### Fire Incidents

The initial results were positive. Over the 12-month period, 11 “real” activations occurred, where alarms prevented the development and spread of fires (see Table 1). In one case, a neighbor was awakened by an alarm, and a little while later sensed intense heat outside her dwelling. She rushed outside to find her neighbor’s dwelling alight and alerted other community members. The fire destroyed four dwellings and damaged another, but her quick response allowed community members to extinguish the fire, preventing it from spreading further. In another case, an inebriated man fell asleep smoking a cigarette, igniting his bedding. His alarm alerted neighbors who extinguished the fire and rescued him before he was seriously burned. There were also several reports of pots of food left unattended on stoves or other cooking-related accidents, where the alarms activated in a timely fashion, and allowed people to extinguish fires before they burned out of control. Many residents reported feeling safer, especially after the alarms successfully averted life and property losses in some dwellings. These successes increased trust in the alarms. In one case, a woman who had taken down her alarm, seeing no value in it, approached the community leader to have it re-installed.

**Table 1:** Details of actual fire alarm activations in Wallacedene temporary relocation area (TRA)

	Timing	Details	Outcome
1	Mid-morning (weekday)	Smoke inside house, with unclear origin	Rescued by neighbor, fire extinguished
2	Weekend	Cooking fire	Alerted homeowner, fire extinguished
3	Evening (weekend)	Cooking left on stove; owner out	Neighbors extinguished fire
4	Late at night	Unknown	Homeowner alerted. Neighbor helped extinguish
5	Unknown	Cooking left on stove	Homeowner out. Neighbors extinguished fire
6	Unknown	Unknown	Homeowner out. Neighbor extinguished fire
7	Late at night	Cooking left on stove. Occupant asleep	Neighbor rescued occupant, extinguished fire
8	Unknown	Cooking left on stove. Occupant asleep	Neighbor rescued occupant, extinguished fire
9	Unknown	Cooking; owner out	Neighbors extinguished fire
10	Unknown	Cooking; owner out	Neighbors extinguished fire
11	Evening	Started by a person smoking in bed	Neighbor rescued occupant, extinguished fire

An unanticipated outcome of the project was that the installation of the alarms and the survey and follow-up monitoring raised levels of fire risk awareness. People reported being more cognizant of the potential for fires and behaviors that could lead to fires. Some said they felt safer knowing that a fire early warning system was in place, while newly arrived residents were eager to have alarms fitted in their dwellings.

### Nuisance Alarms and Challenges

Incidents of nuisance alarms increased over time, gradually desensitizing people to alarm activations. Many households removed their alarms, while some devices were either removed or fell off during renovations or structural changes to dwellings and were never reinstalled. The monitoring suggested that nuisance alarms were caused by oversensitivity to the air quality in dwellings. Qualitative feedback suggested that nuisance activations were commonly triggered by steam or smoke generated during cooking, particularly of oily foods such as meat and when food burned, by people smoking, as well as steam associated with bathing. Other causes included the burning of traditional herbs, and smoke from wood-fired heating devices used inside or near the entrance to dwellings. The monitoring also showed that nuisance alarms were most frequent in smaller dwellings, or where there was no partition between the cooking area and bedroom, where alarms were typically installed. Alarms located towards the top of sloping roofs were also more prone to nuisance activations as smoke and steam rise and naturally accumulate along ceilings. Reports and visual inspections of alarms also suggested that false alarms may have been associated with issues such as dust contamination and insect infestations.

The monitoring identified areas for improvement, including technical modifications to the devices and changes to installation protocols, such as installing alarms on walls instead of ceilings. The findings suggested that modifications such as introducing a mesh housing for the alarm, for example, could prevent particulates and insects from entering devices. Other modifications could include adjusting the interactivity and the timing of the silence feature to make them more suitable to people’s contexts and needs.

## Discussion

In South Africa, informal dwelling fires are rooted in a broader context of poverty, socioeconomic marginalization, and social conditions operating at a national, city, settlement, and household level. Efforts to reduce the incidence and impact of fires ultimately need to tackle these risk accumulation processes. In the shorter-term, active protection mechanisms such as smoke alarms, ideally combined with passive and preventive measures, can help to address the immediate challenge of fires. Given the emphasis on risk reduction globally and in South Africa, and especially the human and material toll of informal dwelling fires in South Africa and comparable environments elsewhere, smoke alarms provide a promising tool for proactively reducing fires and the associated costs for people living in informal settlements, the government, and those providing humanitarian assistance.

The findings of the TRA pilot project suggest that although photoelectric smoke alarms could reduce the risk and spread of fires in informal dwellings, alarms designed for formal spaces cannot be successfully applied wholesale to informal ones. The alarms installed in Wallacedene were subject to a range of technical challenges less experienced in formal dwellings. These included daily exposure to steam and smoke from a variety of sources, compounded by the small size of dwellings, lack of partitioning between rooms, and low roofs; a generally dustier environment; and greater potential for insect infestation. The results indicate that alarms were too sensitive to the air quality in informal dwellings, and nuisance activations increased over time, suggesting that the devices were also affected by the accumulation of dirt and other particulate material, and possibly insects. These findings not only suggest a need for adaptations to make alarms more suitable to conditions in informal dwellings, but also that modifications need to be tested long enough to sufficiently assess their performance over at least 24 months.

The findings also suggest changes to both installation protocols and device modifications. Installing alarms on walls as opposed to ceilings, for example, might decrease nuisance alarms associated with smoke and steam accumulating at ceiling level, while explaining the importance of keeping alarms clean could reduce contamination. Fairly simple technical modifications, such as reducing the sensitivity of the devices or adjusting the interactivity and the timing of the silence feature could improve performance. Introducing a mesh housing could also prevent particulate and insects from entering, while using a pesticide before and after installation might also reduce scope for insect infestation.

More research is needed to identify the most effective changes, but the core challenge in creating alarms that are suitable for informal dwellings is striking the fine balance between enhanced robustness and suitable sensitivity. For example, initial engineering testing of modified smoke alarms following the pilot showed that installing mesh over sensors did reduce the number of nuisance alarms associated with cooking, smoking, and use of smoke-generating energy sources, without significantly reducing activation time (Colclough 2018). However, additional testing is required to determine the correct aperture size for meshes, and changes are needed to accommodate different makes of alarm. Some proprietary smoke alarms already have insect meshes installed, indicating proof of the concept, but these coverings are difficult to keep clean. Innovations, such as a washable housing, could also make alarms more suitable for informal environments.

The critical point is that a more measured approach is needed to ensure that well-intentioned projects are successful in saving lives and livelihoods. It is essential to better understand informal dwellers’ needs and priorities, identify, incorporate, and test refinements to improve the performance of smoke alarms in informal dwellings, and determine their effectiveness over a longer time frame. We also need to provide users with the information and training needed to keep alarms functioning optimally.

At-risk communities have an essential role to play in such processes. Our project in Wallacedene TRA underscored the importance of involving users in the design and implementation of measures to address risk. This is not only necessary to understand their needs and realities, but also to allow them to participate in actively shaping the process and end product. Although community leaders were involved in all aspects of the pilot, the challenges encountered during the monitoring component suggest that more could have been done to engage with the broader community initially and throughout the project to generate a shared understanding and greater sense of ownership. The project’s unanticipated impact on people’s awareness of fire risk nonetheless points to the openings created by even less participatory approaches to generate change—at least in the short term.

Thousands of alarms have already been installed in settlements in Cape Town and its surrounds, but the findings of the Wallacedene project raise questions regarding the effectiveness of these interventions in the long term. There is no silver bullet for addressing a complex problem like informal dwelling fires. Reducing risk will require a bundle of complementary measures. Alongside existing risk reduction measures implemented in the city, such as awareness and education campaigns on reducing fire risk, the installation of alarms could substantially reduce the number and impact of fires in the city, but there is more work to be done to optimize these benefits.

## Conclusion

We cannot eliminate the risk of informal dwelling fires. Inadequate service delivery, the use of dangerous energy sources, such as paraffin stoves and candles, as well as illegal electricity connections that often spark, mean that sources of ignition are plentiful. Owing to the flammability of housing materials, fires also spread rapidly and are difficult to extinguish. Social behaviors, such as alcohol abuse, add additional complexity and are difficult to change. We can, however, reduce the lethality of fires and their magnitude. Early detection of home fires is critical in preventing deaths and injuries, allowing time for people to escape or extinguish a fire. Already widely used in formal dwellings globally, smoke alarms could provide a valuable tool to reduce the frequency and severity of fires in informal dwellings in South Africa, and potentially, in comparable settings elsewhere—even environments such as refugee camps. To be successfully used in informal dwellings, alarm devices need to be modified for use in specific settings. The design of modifications needs to be informed by careful research to understand the needs and priorities of end users. Adaptations need to be tried out in communities for long enough to determine their effectiveness in different settings and over extended periods of time. In keeping with more participatory approaches to disaster risk reduction, it is important that at-risk populations are actively involved in this process. This is necessary to ensure that modifications address not only their needs and priorities, but also provide opportunities for a more inclusive approach to fire risk reduction in which communities (and other stakeholders) collaborate to find solutions to the fire problem.

## Footnotes

1 Detector devices are typically produced on large-scales and the value of units is relatively low. Large-scale rollouts in communities are feasible from a production and unit availability perspective. In developing world contexts, fire safety may often be a lower priority for people compared to other needs, such as safety, health, entertainment, and education. Hence, large-scale rollouts will typically require external funding to achieve significant uptake within an individual community. At the time of the research (2017/2018), each fire alarm cost roughly USD 5, making large-scale rollout of devices challenging in developing world contexts.

2 USD 1 = R 17.79 (8 November 2022).

3 Informal settlements refer to entirely unplanned residential areas that have little or no infrastructure. Most pertinent to this article, they are characterized by informal dwellings (“shacks” or “shanties”), fall outside of building regulations, and have limited or no access to piped water, fire hydrants, or other infrastructure. Formally planned, low-income housing areas are also becoming increasingly “informalized” as residents build makeshift “backyard” dwellings without planning permission to house relatives or to rent out for income. These dwellings are often built of highly flammable materials, lack formal electricity connections, and have residential densities that are even higher than in informal settlements because shacks are packed tightly into walled backyards. Although some consider the term “shack” to be derogatory, the word is used in official government literature for homes, while other terms, such as informal settlement dwellings, wood and iron structures (as per fire department descriptions), and similar expressions, are often ambiguous.

4 Photoelectric and ionization alarms both detect smoke, but in different ways. Ionization-type smoke alarms have a small amount of radioactive material between two electrically charged plates, which ionizes the air and causes current to flow between the plates. When smoke enters the chamber, it disrupts the flow of ions, activating the alarm. Photoelectric smoke alarms, on the other hand, aim a light source into a sensing chamber. When smoke enters the chamber, this reflects light onto a light sensor, triggering the alarm (Mahoney 2022).
